# Prediction of orthostatic hypotension in multiple system atrophy and Parkinson disease

**DOI:** 10.1038/srep21649

**Published:** 2016-02-12

**Authors:** Zhanfang Sun, Dandan Jia, Yuting Shi, Xuan Hou, Xiaosu Yang, Jifeng Guo, Nan Li, Junling Wang, Qiying Sun, Hainan Zhang, Lifang Lei, Lu Shen, Xinxiang Yan, Kun Xia, Hong Jiang, Beisha Tang

**Affiliations:** 1 Department of Neurology, Xiangya Hospital, Central South University, Changsha, Hunan 410008, P. R. China; 2 Key Laboratory of Hunan Province in Neurodegenerative Disorders, Central South University, Changsha, Hunan 410008, P. R. China; 3 State Key Laboratory of Medical Genetics, Central South University, Changsha, Hunan 410008, P. R. China; 4 Department of Geriatrics, Xiangya Hospital, Central South University, Changsha, Hunan 410008, P. R. China; 5 Department of Neurology, the Second Xiangya Hospital, Central South University, Changsha, Hunan 410008, P. R. China; 6 Department of Neurology, the Third Xiangya Hospital, Central South University, Changsha, Hunan 410008, P. R. China

## Abstract

Orthostatic hypotension (OH) is common in multiple system atrophy (MSA) and Parkinson disease (PD), generally assessed through a lying-to-standing orthostatic test. However, standing blood pressure may not be available due to orthostatic intolerance or immobilization for such patients. Systolic blood pressure (SBP) and diastolic blood pressure (DBP) were successively measured in supine, sitting, and standing positions in patients with MSA and PD. Receiver operating characteristic analysis was used to evaluate diagnostic performance of the drops of sitting SBP or DBP. OH and severe OH were respectively regarded as “gold standard”. The drops of SBP in standing position were associated with increased disease severity for MSA and correlated with age for PD. In MSA group, drops in sitting SBP ≥ 14 mmHg or DBP ≥ 6 mmHg had highest validity for prediction of OH, and drops in sitting SBP ≥ 18 mmHg or DBP ≥ 8 mmHg for severe OH. In PD group, drops in sitting SBP ≥ 10 mmHg or DBP ≥ 6 mmHg had highest validity for prediction of OH. The lying-to-sitting orthostatic test is an alternative method for detection of OH in MSA and PD, especially when standing BP could not be validly measured due to various reasons.

Multiple system atrophy (MSA) is an adult-onset, rare, progressive neurodegenerative disease combining parkinsonism, cerebellar ataxia, autonomic dysfunction, and corticospinal disorders. Autonomic dysfunction, mainly including orthostatic hypotension (OH) and genitourinary symptom, is key features of current diagnosis criteria of MSA^1^. OH is defined as a reduction in systolic blood pressure (SBP) ≥ 20 mmHg or diastolic blood pressure (DBP) ≥ 10 mmHg within 3 minutes of standing or head-up tilt to an angle of at least 60 2. A more pronounced reduction ( ≥ 30 mmHg for SBP and/or ≥ 15 mmHg for DBP) is often reported in MSA and is one of the criteria for probable MSA, while diagnosis of possible MSA needs a less severe OH that does not meet the level for probable MSA^1^. In addition, OH is also one of the commonly occurring non-motor symptoms in patients with Parkinson disease (PD). OH may be present at very early stages of PD^3^. OH could lead to gait instability, generalised weakness, and fatigue. Furthermore, OH is an independent risk factor for falls and mortality from many diseases^4^,^5^,^6^.

In clinical practice, however, there are several shortcomings during the measurement of standing BP for patients such as MSA and PD. Firstly, these patients usually cope with severe motor impairment, thus it is difficult for them to keep standing for 3 minutes and the measurement for standing BP is inaccurate due to instability of standing and/or limbs tremor. Additionally, it may be uncomfortable for patients with OH, and even cause life-threatening syncope, during standing for a long time. Passive head-up tilt test (HUT), needing a tilt table with foot board support for BP measurements, may be considered^7^. However, HUT is artificial and does not fully replicate the physiology of active standing because the exercise reflex and the mechanical squeeze on the venous capacitance and arterial resistance vessels are less^8^,^9^. Studies showed that OH may occur more often after tilting than on standing^10^, also suggesting a different physiology between two methods of BP measurement. The aims of this study were to assess the usefulness of drops of sitting blood pressure corresponding to supine blood pressure in predicting the occurrence of OH in patients with MSA and PD.

## Results

### Study Population

During the study period, we prospectively recruited 145 patients with MSA and 213 patients with PD. 12 patients with MSA and 3 patients with PD were excluded because of inability to stand or instability of standing; 2 patients with PD were excluded because of comorbidity of diabetes mellitus; 2 patients with MSA ultimately diagnosed as SCA2 and SCA3 (40 and 75 CAG repeats, respectively for abnormal bands in *ATXN2* and *ATXN3* expansion) were also not included in this study. Therefore, a total of 131 patients with MSA and 208 patients with PD were recruited for this study. The main demographic and clinical characteristics for patients are summarized in Table 1. The patients with MSA included 93 patients with MSA-C and 38 patients with MSA-P. In total, 88 patients and 43 patients fulfilled the criteria for probable MSA and possible MSA, respectively^1^. The majority of patients with PD (64.5%) were in modified H-Y stages 2–4.

### Changes of Blood Pressure from Supine to Sitting and Standing Positions

According to the drops of BP after standing for 3 minutes in this study, OH was common for patients with MSA in contrast to patients with PD after adjusting for gender, age, and duration (61.1% vs 16.8%, *p* < 0.001). Moreover, patients with MSA were more accompanied with severe OH (sOH) after adjusting for gender, age, and duration (35.9% vs 2.4%, *p* < 0.001). There was no significant difference for OH frequency between MSA-P and MSA-C subgroups (26 MSA-P patients and 54 MSA-C patients with OH). Orthostatic hypertension was rare for both patients with MSA and PD. In the correlation analysis, drops of SBP in sitting position was positively correlated with that in standing position for patients with MSA (r_s_ = 0.762, *p* < 0.001) and for patients with PD (r_s_ = 0.618, *p* < 0.001); and a positive correlation was also found for drops of DBP between the two positions (r_s_ = 0.704, *p* < 0.001; r_s_ = 0.634, *p* < 0.001. respectively for patients with MSA and PD). Additionally, the drops of SBP in standing position were more pronounced in the MSA-P than in the MSA-C (standard β coefficient =0.18, *p* < 0.05) and associated with increased disease severity (standard β coefficient =0.17, *p* < 0.05 for UMSARS I), while drops of DBP were only correlated with subtypes of MSA (standard β coefficient =0.18, *p* < 0.05) without other factors such as sex, age, duration, and scores of UMSARS (*p* > 0.05). For patients with PD, drops of SBP in standing position was positively correlated with age (standard β coefficient =0.14, *p* < 0.05) after adjusting for gender, duration, scores of UPDRS, and H-Y stages, but there was no significant correlation between drops of DBP in standing position and these factors.

### Sensitivity and Specificity of Drops of Sitting BP in the Diagnosis of OH

**Figure 1:**
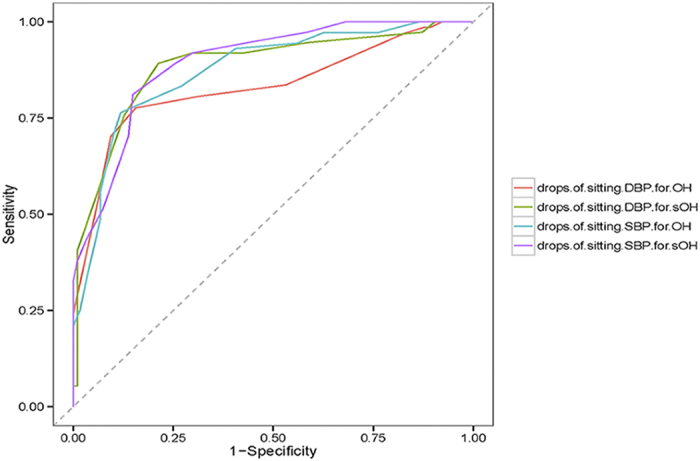
Receiver operating characteristic curves to evaluate the diagnostic performance of the drops of sitting SBP and DBP for OH and sOH in MSA. Area under color curves indicate a moderate predictive value for drops of sitting SBP and DBP to predict OH and sOH (to predict OH, AUC = 0.874 and AUC = 0.831, respectively; to predict sOH, AUC = 0.894 and AUC = 0.885, respectively).

**Figure 2:**
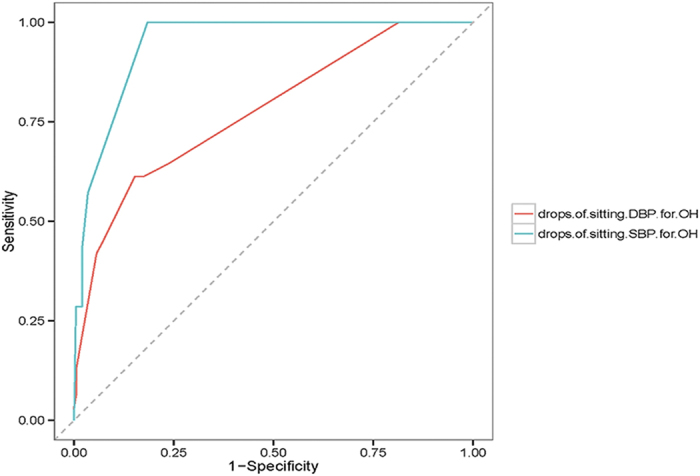
Receiver operating characteristic curves to evaluate the diagnostic performance of the drops of sitting SBP and DBP for OH in PD. Area under color curves indicate a high predictive value for drops of sitting SBP and a moderate predictive value for drops of sitting DBP to predict OH (AUC = 0.946 and AUC = 0.777, respectively)

To explore the validity of drops of sitting BP in the diagnosis of OH, ROC curve was used in patients. When the criteria of OH was regarded as “gold standard”, the diagnostic accuracy of drops of sitting SBP and DBP evaluated by AUC-ROC analysis were 0.874 (95% CI: 0.813–0.934) and 0.831 (95% CI: 0.759–0.904), respectively, for patients with MSA (Fig. 1); 0.946 (95% CI: 0.899–0.993) and 0.777 (95% CI: 0.684–0.870), respectively, for patients with PD (Fig. 2). We further predicted the sOH for MSA with the criteria of sOH as “gold standard”. The diagnostic accuracy of drops of sitting SBP and DBP for sOH were 0.894 (95% CI: 0.837–0.951) and 0.885 (95% CI: 0.815–0.955), respectively (Fig. 1). As shown in Table 2, drops in sitting SBP ≥ 14 mmHg or sitting DBP ≥ 6 mmHg had highest validity for patients with MSA based on the criteria of OH. In the parallel test where drops of sitting SBP or DBP were applied, there was a higher sensitivity (85.0%) and a lower specificity (74.51%) in comparison with the result of either one alone (Table 3). Furthermore, drops in sitting SBP ≥ 18 mmHg or sitting DBP ≥ 8 mmHg had highest validity to predict sOH for patients with MSA with a sensitivity of 89.36% and a specificity of 70.24% in the parallel test (Table 3). Likewise, drops in sitting SBP ≥ 10 mmHg or sitting DBP ≥ 6 mmHg had highest validity to predict OH for patients with PD with a sensitivity of 80.0% and a specificity of 72.83% (Table 2 and Table 3). However, we could not predict sOH using drops of sitting BP due to the fewer patients with sOH in PD group.

## Discussion

OH is commonly associated with neurodegenerative diseases such as PD, MSA and pure autonomic failure^11^,^12^,^13^. OH was reported to affect 9.6–65% of patients with PD and 54–81% patients with MSA, depending on the type of patients and the working definition of OH^14^,^15^,^16^,^17^,^18^,^19^. In our cohort, OH not only appeared more common in MSA than in PD (61.1% vs 16.8%), but also more severe for patients with MSA (35.9% vs 2.4% for sOH), indicating a different pathogenesis of PD and MSA. In general, a degree of damage to the postganglionic sympathetic impairment is suggested as the main cause of OH in PD, including noradrenergic denervation in both cardiac and extracardiac regions and arterial baroreflex failure^16^,^20^. In contrast, OH in MSA is mainly related to loss of sympathetic preganglionic neurons in the intermediolateral column of the thoracolumbar spinal cord^21^. Despite the importance of OH, there is controversy in the literature regarding the methods of detection of OH^19^,^22^. The definition most widely used for OH is based on the consensus of the American Autonomic Society and the American Academy of Neurology^2^. Although it is important to measure the changes of BP in lying-to-standing orthostatic test for diagnosis and treatment of OH, sometimes standing for 3 minutes is intolerable to many individuals and even predisposes to occurrence of syncope. Indeed, studies on OH were often forced to exclude those patients with orthostatic intolerance or immobilization, which would lead to an underestimation of the prevalence of OH; moreover, these patients were commonly accompanied with sOH and may need more attention and immediate treatment. By contrast, the advantages of tilt-table testing are that it is helpful in achieving upright posture in such patients with motor difficulties, but the underlying physiology for tilt-table testing is artificial and special equipment and space are needed.

In the present study, we evaluated the ability of drops of sitting BP corresponding to the supine BP to predict occurrence of OH in patients with MSA and PD. The results showed that the measurement of drops of sitting BP within 3 minutes is a reliable, valid method in such patients. Our findings have significant implications for clinical practice. Using a lying-to-sitting orthostatic test may easily find OH in “previously-neglected” patients with MSA or PD due to orthostatic intolerance or immobilization. Further, delayed OH, which is generally defined as a sustained OH occurring beyond 3 minutes and might reflect an early sympathetic impairment, has recently been reported in patients with MSA or PD^2^,^10^,^17^,^23^. Nevertheless, there is an increased risk of fall or syncope during measurement of standing BP with a delayed standing time (e.g. 15 min, 30 min, or more) in these patients with autonomic dysfunction. In this case, it seems to be more safe and comfortable using this sitting orthostatic test. Moreover, a simple lying-to-sitting orthostatic test would aid in monitoring progression of OH and efficacy of anti-hypotension therapies, and provide an alternative method in the design of clinical trials.

We did not find a significant difference for OH frequency between MSA-P and MSA-C subgroups, which is consistent with a recent natural history study^24^. However, the magnitude of drops of BP appeared to be more pronounced for the MSA-P subgroup. It has been reported that OH is associated with worse prognosis, and that patients with the MSA-P had a shorter survival than those with MSA-C^18^,^25^. Therefore, whether a greater drop in BP was associated with the shorter survival in patients with MSA-P warrants further study. In the PD group, drops of SBP in standing position were merely correlated with age. There were fewer results on magnitude of drops of BP in the course of PD, and a recent study investigating the relationship between drops of BP and duration also obtained similar results^25^, which altogether stressed that OH should be considered in aging patients with PD in spite of a relative lower OH frequency in the PD population^19^.

Our analysis has several limitations. First, the occurrence of OH is known to be influenced by various factors, such as medication use, temperature, time after meals, and comorbidity^26^. Although we controlled these confounding factors (e.g. to measure BP more than 8 hours after medication use, more than 2 hours after meals, and excluding patients with serious comorbidity), the residual effects from these factors might have contributed to OH in our patients and further study with more strict design will be needed. Second, a large heterogeneity between studies on OH has been reported partially due to population heterogeneity and different criteria of OH. Therefore, drops of sitting BP should be explored in other MSA and PD populations using unified criteria of OH. Third, different forms of OH have been described, including initial OH (within the first 30 s), classical OH (within 3 min) and delayed OH (3–45 min)^2^. Whether the lying-to-sitting orthostatic test also applies to the detecting of initial OH and delayed OH should be explored in the future.

## Conclusion

This is the first report to evaluate diagnostic performance of the drops of sitting SBP or DBP for prediction of OH. We demonstrated that the lying-to-sitting orthostatic test is a simple, useful, and valid method to predict occurrence of OH for patients with MSA and PD. The simplicity and safety of the procedure warrant the application of this measurement to individuals, especially to those whose standing BP could not be validly measured due to various reasons.

## Materials and Methods

### Patient Criteria

This sample includes a consecutive unselected cohort of patients with MSA evaluated in 3 study centers specializing in movement disorders in China between May 2010 and March 2015, and of patients with PD in a single center between June 2012 and March 2015. Patients were examined by two or three neurologists who specialized in movement disorders and their care givers were interviewed by members of the MSA team. The diagnosis of patients with MSA was based on the current consensus criteria established by Gilman and colleagues^1^. To exclude the possibility of a diagnosis of spinocerebellar ataxias (SCAs) for patients with MSA, we conducted a mutation analysis for spinocerebellar ataxia subtype 1 (SCA1), SCA2, SCA3, SCA6, SCA7, SCA12, SCA17, and dentatorubral pallidoluysian atrophy, firstly using polymerase chain reaction and 8% polyacrylamide gel electrophoresis, and sequenced the samples showing abnormal bands using “TA cloning” strategy on an automatic sequencer ABI-Prism 3100 (PE Applied Biosystems, Foster City, CA)^27^. The diagnosis of patients with PD fulfilled the United Kingdom PD Brain Bank Criteria^28^. Patients were excluded from our analysis if they met the following criteria: (1) unable to stand for 3 minutes, or apparent instability of standing and/or limbs tremor; (2) a mode of inheritance; (3) less than 8 hours after the last oral medicines such as anti-parkinsonism, anti-depressive, anti-arrhythmic, and anti-hypertensive drugs; (4) have diabetes mellitus or serious dysfunction of heart, liver, and kidney; (5) age of onset less than 30 for MSA and 50 for PD; (6) ultimately diagnosed as SCAs by the mutation analysis; (7) participated in any other clinical trials before giving informed consent.

### Assessments

The study was conducted during clinic hours, duplicating real-world conditions. Blood pressure for each patient was measured in the left arm using auscultatory method with mercury sphygmomanometer (Yuwell, China). The level of measurement was maintained at the level of the heart in all postures to avoid hydrostatic pressure effects of the column. After 10 minutes of supine rest, BP was measured at least twice and averaged to evaluate supine BP. Subsequently, subjects were asked to sit up and then stand up, for 3 minutes, and BP was recorded at 1 and 3 minutes; assistance was provided when that could not be achieved independently. We used the mean of two measurements of BP to evaluate sitting BP and standing BP. A change was calculated as the mean of supine BP measurements minus the averaged sitting BP or standing BP for each patient^2^,^29^. Clinical data including patient demographics and medical history were collected. The clinical stages of MSA were evaluated according to the Unified Multiple System Atrophy Rating Scale (UMSARS), while severity of PD was assessed by the Unified Parkinson’s Disease Rating Scale (UPDRS) and modified Hoehn and Yahr (H–Y) stage at the same visit as for BP measurements.

### Definition

Based on the consensus conference^1^, MSA is divided into two subtypes: MSA with predominant parkinsonism (MSA-P) and MSA with predominant cerebellar ataxia (MSA-C); and into three levels of diagnosis certainty: definite, probable, and possible MSA. OH is defined as a reduction in SBP ≥ 20 mmHg or DBP ≥ 10 mmHg, within 3 minutes of standing, and a reduction in SBP ≥ 30 mmHg or DBP ≥ 15 mmHg for severe OH (sOH)^1^,^2^. Since no formal consensus exists on the definition of orthostatic hypertension, we adopted the most often-used definition, i.e., an increase in orthostatic SBP ≥ 20 mmHg after standing for 3 minutes^29^.

### Ethical approval

The Ethics Committee of Xiangya Hospital of Central South University approved the study and written informed consent was obtained from all subjects. And all the methods were carried out in accordance with the approved guidelines.

### Statistical analysis

All statistical analyses were performed using the Statistical Package for Social Sciences (SPSS version 16.0; SPSS Inc., Chicago, IL) and R language. We assessed the baseline levels in patients with MSA and PD using the χ^2^ test, the t test, or a one-way analysis of variance. We used the Mann-Whitney U test if the data were not normally distributed. To determine whether there was a relationship between the changes of BP in the sitting and standing positions, the Spearman rank correlation analysis was used. We used logistic regression to analyze the difference of OH between MSA and PD. A stepwise linear regression analysis was employed to detect interaction among factors, with drops of SBP and DBP in standing position as the dependent variable after forcing sex, age, duration, scores of UMSARS (or UPDRS, H-Y stages), and subtypes of MSA into the model. Receiver operating characteristic (ROC) curve was used to evaluate the predictive value of change of sitting BP in assessing the OH, with the OH or sOH as reference standard. The area under curve (AUC) was used to indicate the predictive value. An AUC from 0.5 to 0.7 indicates a lower predictive value; AUC from 0.7 to 0.9 indicates a moderate predictive value; AUC > 0.9 indicates a high predictive value. The optimum cutoff value from the ROC curve was determined based on the Youden index^30^.

## Additional Information

**How to cite this article**: Sun, Z. *et al.* Prediction of orthostatic hypotension in multiple system atrophy and Parkinson disease. *Sci. Rep.* **6**, 21649; doi: 10.1038/srep21649 (2016).

## Figures and Tables

**Table 1:** Demographic and clinical characteristics of the total sample.

	MSA (n = 131)	PD (n = 208)
Sex: Men/women	91/40	116/92
Age, y	56.1 ± 7.9	61.7 ± 7.0
Age at onset, y	52.6 ± 7.3	57.3 ± 6.8
Duration, y	3.5 ± 2.1	4.4 ± 3.1
UMSARS I	22.3 ± 9.4	NA
UMSARS II	24.0 ± 8.9	NA
UMSARS VI	3.4 ± 1.2	NA
UPDRS I	NA	1.8 ± 1.5
	NA	13.5 ± 5.6
UPDRS III	NA	27.2 ± 11.6
Hoehn and Yahr stage: n (%)		
1	NA	34 (16.3)
1.5	NA	40 (19.2)
2	NA	58 (27.9)
2.5	NA	33 (15.9)
3	NA	30 (14.4)
4	NA	13 (6.3)
Orthostatic hypotension: n (%)	80 (61.1)	35 (16.8)
Severe orthostatic hypotension: n (%)	47 (35.9)	5 (2.4)
Orthostatic hypertension: n (%)	0 (0)	1 (0.5)
Drops of SBP for sitting position, mmHg	13.4 ± 10.5	2.4 ± 6.2
Drops of DBP for sitting position, mmHg	5.2 ± 7.6	1.2 ± 5.0
Drops of SBP for standing position, mmHg	21.4 ± 15.0	3.7 ± 7.4
Drops of DBP for standing position, mmHg	9.5 ± 11.1	2.0 ± 5.6

Abbreviations: MSA = multiple system atrophy; PD = Parkinson disease;UMSARS = Unified Multiple System Atrophy Rating Scale; UPDRS = Unified Parkinson’s Disease Rating Scale; SBP = systolic blood pressure; DBP = diastolic blood pressure.

Data are mean (SD) unless stated otherwise.

**Table 2:** Sensitivity and specificity of drops of sitting BP in the diagnosis of OH and sOH.

	Patients with MSA								Patients with PD			
	prediction of sitting SBP for OH		prediction of sitting DBP for OH		prediction of sitting SBP for sOH		prediction of sitting DBP for sOH		prediction of sitting SBP for OH		prediction of sitting DBP for OH	
Cutoffs (mmHg)	Sn. %	Sp. %	Sn. %	Sp. %	Sn. %	Sp. %	Sn. %	Sp. %	Sn. %	Sp. %	Sn. %	Sp. %
0	100	13.56	97.01	17.19	100	8.51	97.3	12.77	100	16.92	100	18.64
2	97.22	23.73	83.58	46.88	100	17.02	94.59	41.49	100	60.2	64.52	76.27
4	97.22	27.12	80.6	68.75	100	19.15	91.89	57.45	100	61.69	61.29	82.49
6	97.22	37.29	**77.61**	**84.38**	100	25.53	91.89	70.21	100	67.16	**61.29**	**84.75**
8	94.44	44.07	70.15	90.63	100	31.91	**89.19**	**78.72**	100	80.1	45.16	92.66
10	93.06	59.32	53.73	93.75	97.3	41.49	75.68	87.23	**100**	**81.59**	41.94	94.35
12	83.33	72.88	23.88	100	94.59	56.38	40.54	98.94	57.14	96.52	12.9	99.44
14	**76.39**	**88.14**	20.9	100	91.89	70.21	35.14	98.94	42.86	98.01	6.45	99.44
16	70.83	89.83	16.42	100	89.19	74.47	27.03	98.94	28.57	98.01	3.23	100
18	55.56	93.22	13.43	100	**81.08**	**85.11**	21.62	98.94	28.57	99	3.23	100
20	48.61	93.22	8.96	100	70.27	86.17	13.51	98.94	28.57	99.5	0	100

Abbreviations: MSA = multiple system atrophy; PD = Parkinson disease; OH = orthostatic hypotension; sOH = severe orthostatic hypotension; SBP = systolic blood pressure; DBP = diastolic blood pressure; Sn = sensitivity; Sp = specificity.

The bold indicates sensitivity and specificity corresponding to the optimum cutoffs.

**Table 3:** The cut-off criterion for drops of sitting BP in the prediction of OH and sOH in the parallel test.

	Drops of sitting SBP (or DBP) in patients with MSA		Drops of sitting SBP (or DBP) in patients with PD
	≥ 14 mmHg (or ≥ 6 mmHg)^1^	≥ 18 mmHg (or ≥ 8 mmHg)^2^	≥ 10 mmHg (or ≥ 6 mmHg)^3^
Sn.%	85.00%	89.36%	80.00%
Sp.%	74.51%	70.24%	72.83%

1,2represent the criterion for drops of sitting BP in the prediction of OH and sOH, respectively, for MSA.

3represent the criterion for drops of sitting BP in the prediction of OH for PD.
